# Review of risk factors for human echinococcosis prevalence on the Qinghai-Tibet Plateau, China: a prospective for control options

**DOI:** 10.1186/2049-9957-3-3

**Published:** 2014-01-29

**Authors:** Qian Wang, Yan Huang, Liang Huang, Wenjie Yu, Wei He, Bo Zhong, Wei Li, Xiangman Zeng, Dominique A Vuitton, Patrick Giraudoux, Philip S Craig, Weiping Wu

**Affiliations:** 1 Sichuan Provincial Center for Diseases Control and Prevention, Chengdu, Sichuan, China; 2 Ganzi Prefecture Center for Diseases Control and Prevention, Kangding, Sichuan, China; 3 Institute of Parasitic Diseases, China Center for Diseases Control and Prevention, Shanghai, China; 4 WHO Collaborating Center for Prevention and Treatment of Human Echinococcosis, University Hospital and University of Franche-Comté, 25030 Besançon, France; 5 Department of Chrono-Environment, UMR UFC/CNRS 6249 aff. INRA, University of Franche-Comté, Besançon, France; 6 Cestode Zoonoses Research Group, Bioscience Research Institute and School of Environment and Life Sciences, University of Salford, Great Manchester M5 4WT, UK

**Keywords:** Echinococcosis, Risk factors, Qinghai-Tibet Plateau, Control options

## Abstract

**Objective:**

Echinococcosis is a major parasitic zoonosis of public health importance in western China. In 2004, the Chinese Ministry of Health estimated that 380,000 people had the disease in the region. The Qinghai-Tibet Plateau is highly co-endemic with both alveolar echinococcosis (AE) and cystic echinococcosis (CE). In the past years, the Chinese government has been increasing the financial support to control the diseases in this region. Therefore, it is very important to identify the significant risk factors of the diseases by reviewing studies done in the region in the past decade to help policymakers design appropriate control strategies.

**Review:**

Selection criteria for which literature to review were firstly defined. Medline, CNKI (China National Knowledge Infrastructure), and Google Scholar were systematically searched for literature published between January 2000 and July 2011. Significant risk factors found by single factor and/or multiple factors analysis were listed, counted, and summarized. Literature was examined to check the comparability of the data; age and sex specific prevalence with same data structures were merged and used for further analysis.

A variety of assumed social, economical, behavioral, and ecological risk factors were studied on the Plateau. Those most at risk were Tibetan herdsmen, the old and female in particular. By analyzing merged comparable data, it was found that females had a significant higher prevalence, and a positive linearity relationship existed between echinococcosis prevalence and increasing age. In terms of behavioral risk factors, playing with dogs was mostly correlated with CE and/or AE prevalence. In terms of hygiene, employing ground water as the drinking water source was significantly correlated with CE and AE prevalence. For definitive hosts, dog related factors were most frequently identified with prevalence of CE or/and AE; fox was a potential risk factor for AE prevalence only. Overgrazing and deforestation were significant for AE prevalence only.

**Conclusion:**

Tibetan herdsmen communities were at the highest risk of echinococcosis prevalence and should be the focus of echinococcosis control. Deworming both owned and stray dogs should be a major measure for controlling echinococcosis; treatment of wild definitive hosts should also be considered for AE endemic areas. Health education activities should be in concert with the local people’s education backgrounds and languages in order to be able to improve behaviors. Further researches are needed to clarify the importance of wild hosts for AE/CE prevalence, the extent and range of the impacts of ecologic changes (overgrazing and deforestation) on the AE prevalence, and risk factors in Tibet.

## Multilingual abstract

Please see Additional file 1 for translations of the abstract into the six official working languages of the United Nations.

## Background

Human cystic echinococcosis (CE) and alveolar echinococcosis (AE) are caused by the larval stage of *E. granulosus* and *E. multilocularis*, respectively. Transmission of CE and AE to humans is by unintentional consumption of parasite eggs excreted in the feces of the definitive hosts such as dogs and foxes. Geographically, CE has a universal distribution, while AE is confined to the northern hemisphere. In humans, infection results in a metacestode in the liver mostly. Chemotherapy is required continuously for many years, or even for the entire lifetime. In the absence of treatment, the disease is fatal. AE mortality could reach 94% and above if untreated within 10 years [1,2]. It has been acknowledged as one of the world’s most lethal parasitic zoonoses [3].

**Figure 1:**
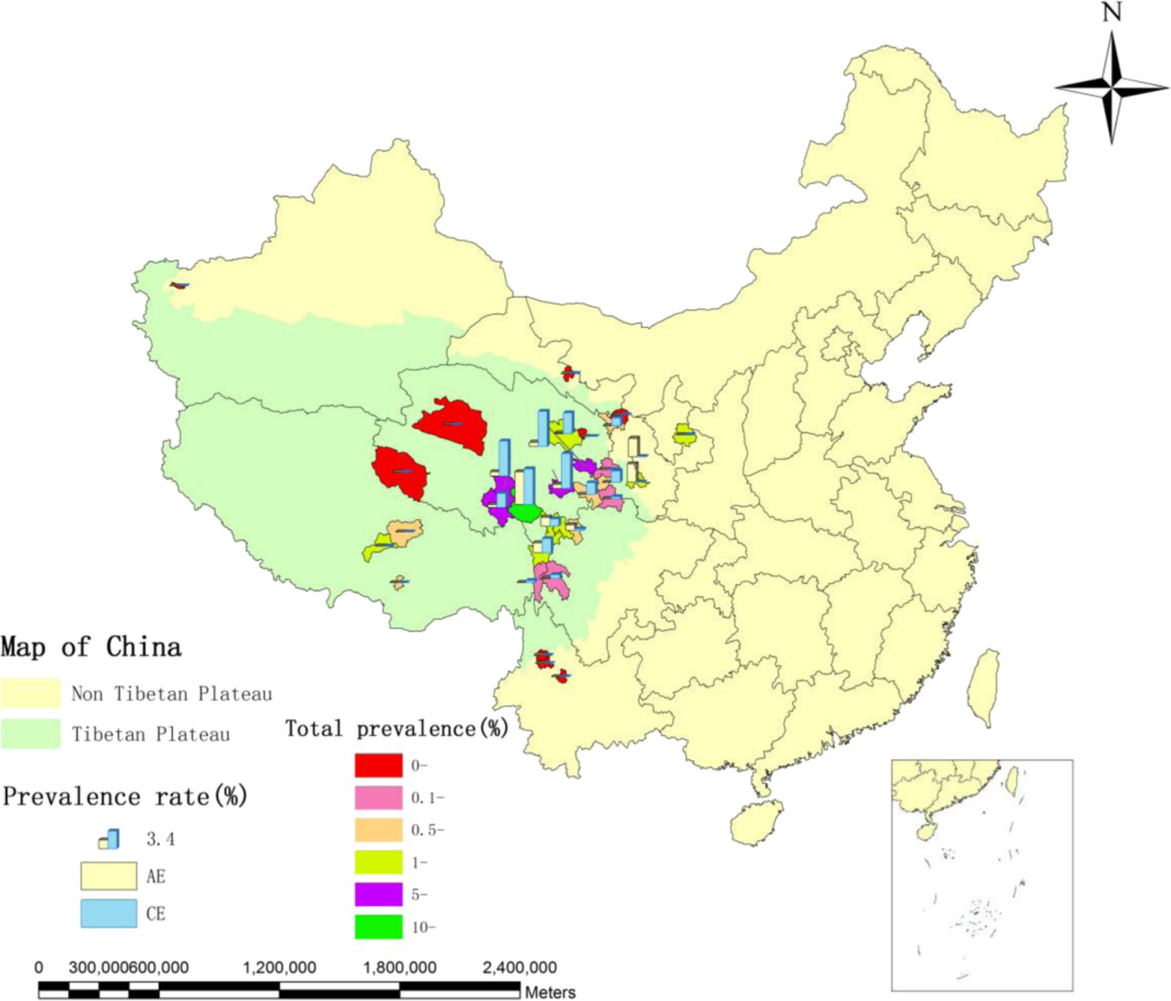
**Human echinococcosis prevalence on the Qinghai-Tibet Plateau. ***Note:* within the legend, the right blue bar indicates 3.4% of CE prevalence. *References:* 7, 10–13, 15–39.

The World Health Organization (WHO) listed echinococcosis, including both CE and AE, as a neglected tropical disease in 2010 [4]. The estimated worldwide human burden of CE is 285,407 (95% confidence interval [CI], 218,515–366,133) DALYs [5], and the figure for AE is 666,434 DALYs (CIs 331,000–1.3 million) [6]. China is responsible for 40% of the global CE DALYs [5] and 91% of global AE DALYs [6]. In western China, 380,000 patients were estimated by a nationwide sampling survey conducted from 2001 to 2004 [7]. The figure indicates that western China is a highly-endemic area of echinococcosis worldwide. The survey estimated that the prevalences of echinococcosis in Tibet, Tibetan communities in Sichuan, and Qinghai Province were 2.76%, 2.33%, and 1.91%, respectively. These figures were the top three in the nation, and indicated Qinghai-Tibet Plateau to be the most endemic area in China (see Figure 1).

The Qinghai-Tibet Plateau is located at 26°00′12″N ~ 39°46′50″N and 73°18′52″E ~ 104°46′59″E in western China. It covers most of the Tibet Autonomous Region and the Qinghai Province, as well as part of the Sichuan Province, the Gansu Province, the Xinjiang Uygur Autonomous Region, and the Yunnan Province. A part of the Plateau is bordered to the south by the Himalayan range, to the north by the Kunlun range, to the northeast by the Qilian range, to the east by the Hengduan Mountains, and to the west by the Pamir Mountains. There are some 155 counties/cities located in the Plateau, and some 61 counties/cities at the edge of the Plateau. The estimated population was 1,204 million [8].

Two forms of echinococcosis, CE and AE, are highly endemic on the Qinghai-Tibet Plateau [7]. The domestic lifecycle and sylvatic lifecycle were assumed for *E. granulosus* and *E. multilocularis*[9] respectively, and indicated that the risk factors could be very complicated and diversified. To curb the transmission of the disease, the Chinese government is increasing the financial support for the control of echinococcosis. The resources allocation will largely depend on the control options decided on by keeping the significant risk factors in mind. Therefore, it is high time to review and identify the risk factors for echinococcosis on the Qinghai-Tibet Plateau, where the transmission AE and/or CE is very dynamic [7,10-13]. The findings could help policymakers design more targeted control strategies.

## Review

### Methods

#### Selection criteria

Literature with the following characteristics was included: 1. Diagnosis of human echinococcosis for community-based studies was based on positive findings by abdominal ultrasound examinations and complemented by confirmatory serological tests, as WHO suggested [14]; diagnosis for hospital-based studies were dependent on abdominal ultrasounds, X-rays, computed tomography examinations, and complemented by confirmatory serological tests. 2. Risk factors were linked to human prevalence. 3. Literature had to refer to an actual study rather than be a review of a study/studies.

### Search strategy

**Figure 2:**
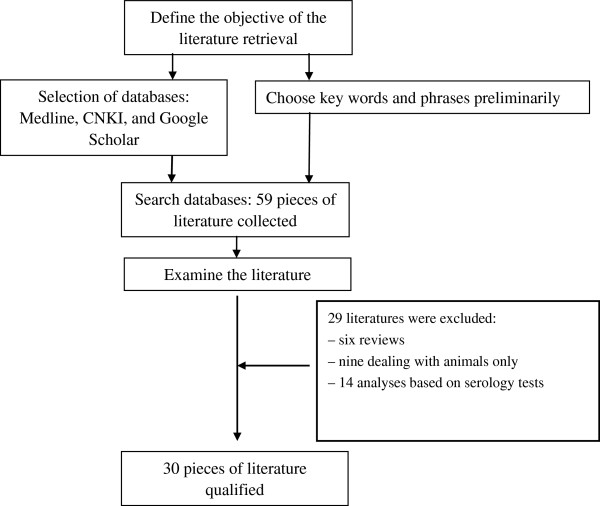
Strategy for literature retrieval.

Medline, CNKI, and Google Scholar were searched for literature which was published between January 2000 and July 2011. Search terms included one word and/or phrase from each of following three categories: the name of the disease, including hydatidosis, hydatid disease, echinococcosis, and echinococcus; risk factors, including social, economy, religion, risk, behaviors, income, hygiene, and Buddhism; location, including Tibet, Qinghai, Qinghai-Tibet Plateau, Tibet Plateau, western China, Sichuan, Gansu, Yunnan, and Xinjiang. The languages of the literature were restricted to Chinese and English. Figure 2 shows a visualized strategy for the literature retrieval.

### Data analysis

Significant risk factors found by single factor and/or multiple factors analysis were listed, counted, and summarized. The significant level of risk factors was P < 0.05 for both single factor and multiple factors analyses. Age specific prevalence with the same age span in each age group was merged from the data found in the literature. With the merged data, a trend figure of age specified prevalence was produced by Excel; an R-squared value was calculated and the linearity of the trend was tested. Sex specific data from the literatures were merged; Chi-square was used to compare the merged prevalence between males and females; 95% confidence intervals were calculated for the sex specific prevalence.

## Results

In total, 30 studies were found to address risk factors related to echinococcosis prevalence on the Qinghai-Tibet Plateau in the past decade [7,10-13,15-39]. Of those, ten, four, twelve, three, seven, and one were carried out in Qinghai, Tibet, Sichuan, Yunnan, Gansu, and Xinjiang, respectively (some studies covered several provinces/regions). The studies for human prevalence were community-based except for two case studies conducted in Tibet, which were hospital based [15,32]. The participants of the community-based studies were all self-selected. Eight studies analyzed the risk factors for CE prevalence [15,16,23-26,32], 13 studied risk factors for AE prevalence [10,13,16,17,20,21,23,25],[26,34-37], and 13 analyzed the risk factors for both AE and CE [7,11,12,18,19,23,25,27-30],[38,39].

The studied risk factors were demographics, lifestyle, hygiene practices, drinking water source, slaughter and viscera disposal practices, animal ownership and dog related practices, small mammal indices, and land use practices (see Table 1).

**Table 1:** Risk factors for human echinococcosis prevalence on the Qinghai-Tibet Plateau

**Risk factors**	**Number of studies which found the factor being significant by single factor analysis and references**			**References and locations of studies**
	**Echinococcosis**	**CE**	**AE**	
***Demographics***				
Increasing age	7(7,11,12*, 27,30,38, 39)	8(12,15, 16*,18,23,25,26*,31)	8(12, 16*,20,21,23,25, 26*,36*)	7(Sichuan, Yunnan, Gansu, Qinghai, Xinjiang, Tibet), 11(Qinghai), 12(Qinghai), 15(Tibet), 16(Sichuan), 18(Qinghai), 20(Gansu), 21(Gansu), 23(Qinghai), 25(Qinghai), 26(Sichuan), 27(Sichuan), 30(Qinghai), 31(Gansu), 36(Sichuan), 38(Qinghai), 39(Sichuan)
Female	9(7,11,12*,22,27,28,30,38,39)	1(23)	6 (16,20,21,23,25,36*)	7(Sichuan, Yunnan, Gansu, Qinghai, Xinjiang, Tibet), 11(Qinghai), 12(Qinghai), 16(Sichuan), 20(Gansu), 21(Gansu), 22(Yunnan), 23(Qinghai), 25(Qinghai), 27(Sichuan), 28(Qinghai), 30(Qinghai), 36(Sichuan), 38(Qinghai), 39(Sichuan)
Herding population	7(12*,19,25,27,30,32,38)	3 (15,16,23)	1(36)	12(Qinghai), 15(Tibet), 16(Sichuan), 19(Gansu), 25(Qinghai), 23(Qinghai), 27(Sichuan), 30(Qinghai), 32(Tibet), 36(Sichuan), 38(Qinghai)
Tibetan population	2(12,28)		1(36)	12(Qinghai), 28(Qinghai), 36(Sichuan)
Farming population	1(32)		2(20, 21)	20(Gansu), 21(Gansu), 32(Tibet)
Lama	1(28)			28(Qinghai)
Nomadic	1(12*)	1(23)	1(16)	12(Qinghai), 16(Sichuan), 23(Qinghai)
Lower income	1(12)		1(36)	12(Qinghai), 36(Sichuan)
Limited school education	2(12,27)	1(23)	1(36)	12(Qinghai), 27(Sichuan), 23(Qinghai), 36(Sichuan)
*Animal ownership*				
Livestock ownership	1(12*)	1(16*)	2(16*, 36)	12(Qinghai), 16(Sichuan), 36(Sichuan)
Dog ownership		1(23)	1(23)	23(Qinghai)
Total number of owned dogs			1(10)	10(Gansu)
Keeping two or more dogs			2(20,21)	20(Gansu), 21(Gansu)
Longer period of dog ownership		1(31)		31(Gansu)
Stray dogs present		1(23)		23(Qinghai)
*People’s behaviors related to dogs*				
Playing with dogs		1(16*)	4 (16*, 20, 21, 36*)	16(Sichuan), 20(Gansu), 21(Gansu), 36(Sichuan)
Leaving dogs untied			2(20,21)	20(Gansu), 21(Gansu)
Allowing dogs to sleep indoors at night	1(12*)			12(Qinghai)
Feeding dogs with viscera	1(12)	1(23)		12(Qinghai), 23(Qinghai)
Using dog feces as fertilizer			2(20, 21)	20(Gansu), 21(Gansu)
Fox skin products ownership			1(36*)	36(Sichuan)
*Hygiene-related behaviors*				
Ground water as a drinking water source	1(12)	2(23, 31)	2(16, 36*)	12(Qinghai), 16(Sichuan), 23(Qinghai), 31(Gansu), 36(Sichuan)
Never boiling water before consumption	1(12)			12(Qinghai)
Not washing hands before eating			2(23, 36)	23(Qinghai), 36(Sichuan)
Not protecting food from flies		1(16)	2(16, 36*)	16(Sichuan), 36(Sichuan)
*Ecological factors*				
Density indices of voles			4(10,13,17,35)	10(Gansu), 13(Sichuan), 17(Gansu), 35(Sichuan)
Deforestation			2(10,17)	10(Gansu), 17(Gansu)
Overgrazing			4(13,26*, 35,37)	13(Sichuan), 26(Sichuan), 35(Sichuan), 37 (Sichuan, Qinghai)

*Note:* “*”indicates that it was significant by multiple factors analysis also.

For CE and/or AE together, increasing age [7,11,12,15,16,18,20-23,25-27],[30,31,36,38,39], being a female [7,11,12,20-23,25-27,30,36,38],[39], and coming from a herding population [12,15,19,23,25-27,30,32,36],[38] were the most significant demographic risk factors. In terms of behavioral factors, dog related factors were mostly correlated with CE and/or AE prevalence [10,12,16,20,21,23,31,36]. Longer periods of dog ownership [31] and stray dogs [23] were significant for CE prevalence only. In terms of hygiene, employing ground water as the drinking water source [12,23,31,36] was significantly correlated with CE and/or AE prevalence [36]. Overgrazing [13,26,35,37] and deforestation [10,17] were significant for AE prevalence only.

Only one study [12] applied multiple factors analysis to identify risk factors for echinococcosis prevalence; the analysis included risk factors in the categories of demographics, lifestyle, slaughter and viscera related practices, animal ownership and dog related practices, water source, and hygiene practices. It found that increasing age, being a female, yak and/or sheep ownership, and allowing dogs to sleep indoors at night to be significant risk factors. For CE prevalence, two studies [16,26] applied multiple stepwise logistic regression. One analysis [16] included risk factors of demographics, lifestyle, slaughter and viscera related practices, animal ownership and dog-related practices, water source, and hygiene practices. It found increasing age, yak and/or sheep ownership, and playing with dogs to be significant risk factors. Another study [26] included age, sex, and the area of fenced pasture into a multiple stepwise logistic regression, and identified increasing age to be significant. To identify risk factors for AE prevalence, three studies [16,26,36] applied multiple stepwise logistic regressions, which included age, sex, and other factors. Increasing age was identified by three studies [16,26,36]; playing with dogs was found by two studies [16,36]; and factors identified by one study included being a female [36], yak and/or sheep ownership [16], fox skin products ownership [36], ground water as the drinking water source [36], overgrazing [26], and no protection of food from flies [36]. The significance of overgrazing was further supported by studies on the transmission of *E. multilocularis*[35,40].

**Figure 3:**
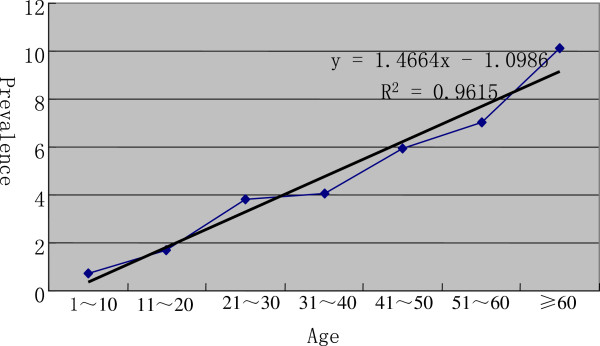
**Trends of age specified prevalence of human echinococcosis on the Qinghai-Tibet Plateau. ***Notes*: 1. Data source: 4,7, and 24. 2. Sample size was 23,785. 3. Linearity test: F = 124.9, P = 0.0001.

The significance of increasing age and being a female was further supported by analyzing merged comparable data from three studies [7,11,29] conducted in Sichuan and the Qinghai Province. A strong trend of linearity was found between increased prevalence and increased age (F = 124.9, P = 0.0001, R^2^ = 0.9615, sample size = 23,785) (see Figure 3). By using a Chi-square test of merged data from two studies in Tibet [7], Sichuan Province [7], and Qinghai Province [7,11], it was found that female prevalence (4.22%, 95% CI: 4.05–4.39%, sample size = 13927) was significantly (χ^2^ =39.06, P < 0.01) higher than that of male prevalence (2.81%, 95% CI: 2.67–3.00%, sample size = 13009).

## Discussion and conclusion

The reviewed studies [7,10-13,15-39] covered majority areas of the Qinghai-Tibet Plateau. However, most studies were done in Sichuan, Qinghai, and Gansu, while only three studies were done in Tibet [7,15,32]. Another important finding was that the studies were mostly done in highly-endemic areas with a prevalence of more than 1%, except for three studies which were conducted in the Yunnan Province and one study in Xinjiang, where the prevalences were reported to be 0.03% and 0.19%, respectively.

The review identified that the Tibetan herdsman population, the old and females in particular, is at a significant higher risk of infliction by echinococcosis. Therefore, this population should be the focus of the control strategies. Dog- and fox-related risk factors for AE prevalence were studied carefully by several studies. Dog-related risk factors were found to be mostly significant [10,20,21,23,26,31,36], while only one fox-related risk factor was found significant [36]; the factor was fox skin products ownership. On the Plateau, several studies found that both foxes and dogs (definitive hosts) [12,19,30,34,38,41] had a high prevalence of *E. multilocularis*. However, studies in Sichuan [26,27,36] and Gansu [20,21] could not find that the fox hunting population had a significant higher AE prevalence compared with other populations; no other studies tried to link foxes to AE prevalence statistically on the Plateau. These findings indicated that dogs may serve as a more important transmission source for human AE. This is a significant finding for AE prevalence. This finding is very different from the situation in Europe and Japan, where foxes were typically considered as a major transmission source of human AE [10,42,43]. Due to the significance of the fox skin products ownership, the role and the extent of foxes in the transmission of AE to humans should be the focus of future research.

Presence of stray dogs was significant for CE prevalence [23], but it may also have a role in advancing AE prevalence. A study found that the *E. multilocularis* prevalence in owned dogs was statistically associated with the number of stray dogs [40]. The killing of stray dogs is not possible due to local Buddhist beliefs that prohibits killing [12,39]. Therefore, the feasible control measures with priority on the Plateau should be treating both domestic and stray dogs with Praziquantel, dosing of foxes, and limiting dogs’ access to infected viscera, when applicable. Key behaviors, such as not washing hands before eating and playing with dogs, should change. A health education strategy in concert with the population’s education background and local languages is a must to effectively disseminate knowledge in order to help improve key behaviors.

*E. granulosus*, whose lifecycle is considered to be typically domestic [9], could possibly have a wild ungulate component on the Plateau; it was reported that wild ungulates died in large numbers due to cold and hunger in early spring [44]. Due to increasing protection by national laws and policies, the number of wild ungulates has been increasing consistently in recent years; for example, in Maduo county, Qinghai Province, the number of Tibetan gazelle, which live in the area between the altitude of 3,000 m to 5,000 m, increased from 25,000 in 2000 to more than 40,000 in 2010. The number of Tibetan wild ass (*Equus kiang*) and blue sheep (*Pseudois nayaur*) had also increased drastically [45]. These animals are ungulates that are susceptive to *E. granulosus*[9]. The *E. granulosus* infection rate was reported to be 6.42% (21/327) for blue sheep and 6.57% (13/198) for Tibetan gazelle in the Qinghai part of the Plateau [46]. Definitive hosts, including foxes and wolves, were found to be infected with *E. granulosus*[12,28,38]. A recent study indicated a possible contribution to the dogs’ infection in early spring by dead wild ungulates in the Sichuan part of the Plateau [47]. Therefore, the role of wild hosts in the transmission of *E. granulosus* cannot be ignored, and further researches are needed.

Multiple factors regression is considered to be a far more accurate test than a single factor test; multiple factors regression returns results for the combined influence of all independent factors (variables) on the dependent factor, as well as the individual influence of each independent factor while controlling for the other independent factors [48]. The significant factors identified by multiple factors analyses were increasing age [12,26,36], being a female [12,36], herding population [12], yak and/or sheep ownership [12,16], allowing dogs to sleep indoors at night [12], playing with dogs [16,36], fox skin products ownership [36], ground water as a drinking water source [36], overgrazing [26], and no protection of food from flies [36].

Overgrazing and deforestation were found to be significant for AE prevalence only; the former was studied in the Sichuan Province [13,26,35,40] and assumed in Qinghai [30]; the latter was found significant in the eastern fringe of the Plateau in the Gansu Province [17]. Overgrazing and deforestation are considered major ecological phenomena in western China [49-51]. Both risk factors deserve further research to understand their role in general, and in promoting the transmission of *E. multilocularis* and consequently advancing AE prevalence, as well as their implications for control options.

In conclusion, echinococcosis was identified as a priority for control and research globally, and in China [4,7,52-54]. Deworming both owned and stray dogs should be a major measure for controlling echinococcosis on the Plateau; treatment of wild definitive hosts should also be considered for AE-endemic areas. Health education activities should be in concert with the local population’s education background and local languages in order to help improve behaviors. Further researches are needed to clarify the importance of wild hosts for the AE/CE prevalence, the impact of ecologic changes (overgrazing and deforestation) on the AE prevalence, and risk factors in Tibet.

## Competing interests

The authors declare that they have no competing interests.

## Authors’ contributions

WQ, WW, ZB and LW conceived the review concept, carried out the literature search, developed the structure for the manuscript, and drafted the paper. Other authors participated to organize the draft sections, co-wrote sections of the draft, and edited the overall manuscript. All authors read and approved the final version of the manuscript before its submission to IDP.

## Supplementary Material

Additional file 1 Multilingual abstracts in the six official working languages of the United Nations. Click here for file
