# Supplementary material for: Review of risk factors for human echinococcosis prevalence on the Qinghai-Tibet Plateau, China: a prospective for control options
Source: Infect Dis Poverty. 2014 Jan 29;3:3. doi: 10.1186/2049-9957-3-3 (PMC3910240; doi:10.1186/2049-9957-3-3)
Supplement: Additional file 1 — Multilingual abstracts in the six official working languages of the United Nations. [file 2049-9957-3-3-S1.pdf]

Translation of the abstract into the six official working languages of the United Nations

استعراض عوامل الخطر المتسببة في انتشار داء المشوكات لدى الإنسان في هضبة التبت بالصين: دراسة تمهيدية لخيارات مكافحة المرض

وانج كيان، هوانج يان، هوانج ليانج، يو وينجي، هي وي، إل زونج بو، لي وي، زنج إكسيانجمان، دومينيك إيه. فويتون، باتريك جيرودو، فيليب إس. كريج، وو ويبينج

الملخص

الهدف: يعتبر مرض المشوكات أحد الأمراض الرئيسية الناشئة عن طفيليات حيوانية تصيب الإنسان ويشكل أهمية للصحة العامة في الصين الغربية. ففي عام 2004، قدرت وزارة الصحة الصينية إصابة 380،000 مريض في المنطقة بالمرض. وتعتبر المشوكات السنخية والمشوكات الكيسية على السواء من أكثر الأمراض المستوطنة في هضبة تشنغهاي التبت. لذلك، عملت الحكومة الصينية على زيادة الدعم المالي لمكافحة الأمراض في هذه المنطقة. ومن ثم، كان من الأهمية بمكان تحديد عوامل الخطر الرئيسية للأمراض من خلال مراجعة الدراسات التي أجريت في العقد الماضي لمساعدة صناع القرار على تصميم استراتيجيات المكافحة المناسبة.

الطرق: كان أول ما تم تحديده هو معايير اختيار المؤلفات التي يجب مراجعتها. وكان يجري بانتظام البحث في ميدلين والبنية التحتية المعرفية الوطنية الصينية والباحث العلمي لجوجل لانتقاء المؤلفات المطبوعة ما بين يناير 2000 ويوليو 2011. وقد تم إدراج عوامل الخطر الرئيسية التي وجدت سواء كانت تحليل لأحد العوامل و/أو تحليل لعدة عوامل وحصرها وتلخيصها. وتم فحص المؤلفات للتحقق من إمكانية مقارنة البيانات؛ وتم دمج مدى انتشار المرض في سن وجنس معين بنفس هياكل البيانات لاستخدامها في إجراء مزيد من التحليل.

النتائج: أجريت دراسة على مجموعة افتراضية متنوعة من عوامل الخطر الاجتماعية والاقتصادية والسلوكية والبيئية في الهضبة، وكان أكثر المعرضين للخطر الرعاة التبتيين وكبار السن والإناث على وجه الخصوص. وبتحليل البيانات المقارنة التي تم دمجها، وجد أن أعلى معدل انتشار كان بين الإناث، مع وجود علاقة تزامن خطية إيجابية بين انتشار داء المشوكات والتقدم في العمر. فبالنسبة لعوامل الخطر السلوكية، ارتبط اللعب مع الكلاب غالباً بانتشار المشوكات الكيسية و/أو المشوكات السنخية. وبالنسبة لعامل النظافة الصحية، فقد ارتبط، إلى حد كبير، استخدام المياه الجوفية كمصدر لمياه الشرب بانتشار المشوكات الكيسية والمشوكات السنخية. أما العوائل المحددة، فقد وجدت، في كثير من الأحيان، عوامل ذات صلة بالكلاب كسبب لانتشار المشوكات الكيسية و/أو المشوكات السنخية؛ بينما كانت الثعالب عامل خطر محتمل لانتشار المشوكات السنخية فقط. وكان الرعي الجائر وإزالة الغابات أيضاً سبباً هاما لانتشار المشوكات السنخية وحدها.

النتيجة: كانت مجتمعات الرعاة التبتيين أكثر المجتمعات تعرضاً لخطر انتشار داء المشوكات. لذلك، ينبغي أن تكون هذه المجتمعات موضع التركيز للسيطرة على داء المشوكات. كذلك، ينبغي أن يكون التخلص من الديدان لدى الكلاب المملوكة والضالة على حد سواء أحد الإجراءات الرئيسية للسيطرة على المشوكات؛ وينبغي أيضا وضع علاج العوائل البرية المحددة في المناطق الموبوءة بالمشوكات السنخية في الاعتبار. ومن ثم، ينبغي أن تكون أنشطة التثقيف الصحي متناسقة مع خلفيات تعليم المجتمع المحلي ولغتهم لكي تستطيع تحسين السلوكيات.

ويجب إجراء مزيد من الأبحاث لتوضيح أهمية العوائل البرية المتسببة في انتشار المشوكات السنخية/المشوكات الكيسية، ومدى تأثير التغيرات البيئية (الرعي الجائر وإزالة الغابات) ونطاقها على انتشار المشوكات السنخية، وعوامل الخطر في منطقة التبت.

Translated from English version into Arabic by Noha Mostafa, through

青藏高原包虫病流行风险因素及其防控策略

王谦，黄燕，黄亮，喻文杰，何伟，钟波，李伟，曾祥嫚，多米尼克.维通，帕特克.热合都，菲利普.克瑞格，伍卫平

摘要

引言: 包虫病是中国西部具有重要公共卫生意义的寄生虫人兽共患病。 2004 年，中国卫生部估计该区域有 38 万病人。青藏高原是囊型包虫病和泡型包虫病的高度流行区。在过去数年中，中国政府不断增加青藏高原包虫病防治经费。因此，回顾过去 10 年该区域关于流行风险因素的研究，确定重要的流行风险因素，帮助决策者设计适当的防控策略是十分重要的。

方法: 首先确定文献回顾的文献选择标准。通过系统地搜索美国国立医学图书馆国际性综合生物医学信息书目数据库、CNKI (国家知识基础设施)及谷歌学术搜索，寻找发表于 2000 年 1 月到 2011 年 7 月之间的中英文文献。对文献中通过单因素或者多因素分析发现的具有统计学显著意义的因素进行列表、计数和总结。检查文献发表的数据可比性，对具有相同数据结构的年龄别和性别患病率数据进行合并，对这些合并的数据进行进一步分析。

结果：这些文献对一系列假定的青藏高原社会、经济、行为和生态风险因素进行了研究。研究表明，藏族牧民是患病风险最高的人群，高年龄人群和女性患病风险特别高。对合并的数据分析表明，女性患病率显著较高，年龄和患病率之间存在一个线性正相关。在行为风险因素方面，耍犬是最频繁出现的包虫病、囊型包虫病和泡型包虫病流行风险因素。在卫生方面，地面水作为饮用水源与包虫病、囊型和泡型包虫病显著相关。在终宿主方面，无论对泡型包虫病还是囊型包虫病，最频繁出现的显著风险因素均与犬有关; 狐狸是一个潜在的泡型包虫病流行风险因素。草原过牧和森林采伐对泡型包虫病的流行具有显著意义。

结论：藏族牧区包虫病流行风险最高，是包虫病防控重点区域。对家、野犬驱虫是包虫病防控主要措施；在泡型包虫病流行区，也应该对野生终宿主进行驱虫。健康教育应该与当地人群的教育背景、语言紧密结合，以达到改进行为目的。未来研究需要进一步明晰野生终宿主对包虫病流行的意义、生态学改变（草原过牧和森林采伐）对包虫病流行影响的程度和范围、以及西藏区域的流行风险因素。

Translated from English version into Chinese by WANG Qian

Examen des facteurs de risque de prévalence de l'échinococcose humaine sur le plateau de Qinghai-Tibet en Chine : étude prospective des méthodes de lutte contre l'infection

WANG Qian, HUANG Yan, HUANG Liang, YU Wenjie, HE Wei, L ZHONG Bo, LI Wei, ZENG Xiangman, Dominique A. VUITTON, Patrick GIRAUDOUX, Philip S. CRAIG, WU Weiping

Résumé

Objectif : L'échinococcose est une importante zoonose parasitaire relevant de la santé publique dans l'ouest de la Chine. En 2004, le Ministre de la Santé chinois estimait que la maladie touchait 380 000 patients dans cette région. Les formes échinococcose alvéolaire (EA) et échinococcose cystique (EC) sont fortement co-endémiques du plateau Qinghai-Tibet. Au cours des dernières années, le gouvernement chinois a renforcé le soutien financier pour la lutte contre ces maladies dans cette région. Il est donc très important d'identifier les principaux facteurs de risque de ces maladies en examinant les études réalisées dans la région au cours des dix dernières années, afin d'aider les responsables politiques à concevoir des stratégies de lutte adaptées.

Méthodes : Des critères de sélection de la littérature à examiner ont d'abord été définis. Des recherches systématiques ont été effectuées sur Medline, CNKI (China National Knowledge Infrastructure), et Google Scholar pour trouver la littérature publiée entre janvier 2000 et juillet 2011. Les principaux facteurs de risque découverts au cours des analyses à facteur unique et/ou des analyses à facteurs multiples ont été listés, comptés et résumés. La littérature a été examinée afin de vérifier la comparabilité des données ; les prévalences en fonction de l'âge et du sexe utilisant les mêmes structures de données ont été regroupées et utilisées pour effectuer des analyses plus approfondies.

Résultats : Un grand nombre de facteurs de risques sociaux, comportementaux et écologiques supposés ont été étudiés pour cette région. Les personnes les plus à risque sont les bergers tibétains, en particuliers les femmes et les personnes âgées. À l'analyse des données comparables regroupées, il est apparu que les femmes présentaient une prévalence nettement supérieure, et qu'il existait une relation linéaire positive entre la prévalence de l'échinococcose et le vieillissement. En ce qui concerne les facteurs de risque comportementaux, le fait de jouer avec des chiens est fréquemment associé à une prévalence d'EC et/ou d'EA. En matière d'hygiène, c'est

principalement l'utilisation des eaux souterraines comme eau potable qui est associée à une prévalence d'EC et EA. Chez les hôtes définitifs, les chiens sont le facteur le plus fréquemment identifié de la prévalence d'EC et/ou EA ; le renard représente un facteur de risque potentiel de la prévalence d'EA uniquement.

Conclusion : Les communautés de bergers tibétains sont exposées à un risque élevé de prévalence d'échinococcose, et c'est sur elles que doivent se concentrer les efforts de lutte. La vermifugation des chiens domestiques et errants est une des mesures principales de la lutte contre l'échinococcose ; il faut également envisager de traiter les hôtes définitifs sauvages dans les zones où l'EA est endémique. Des activités d'éducation à la santé adaptées au niveau d'instruction et à la langue des populations locales doivent être mises en place afin de permettre une amélioration des comportements. Des recherches complémentaires sont nécessaires pour clarifier l'importance des hôtes sauvages dans la prévalence d'EA/EC, l'étendue et la portée des conséquences des changements écologiques (surpâturage et déforestation) sur la prévalence d'EA, ainsi que les facteurs de risque du Tibet.

Translated from English version into French by MarinaJ, through

TRANSLATORS
WITHOUT BORDERS

Обзор факторов риска эхинококкоза у человека в районе Цинхай-Тибетского нагорья, Китай: потенциальные контролирующие мероприятия

ВАНГ Квиан, ХУАНГ Ян, ХУАНГ Лианг, Ю Венджи, ХЕ Вей, ЭЛЬ ДЖОНГ Бо, ЛИ Вей, ДЖЕНГ Ксиангман, Доминик А. ВУИТТОН, Патрик ЖИРОДУ, Филипп С. КРЕЙГ, ВУ Вейпинг

Резюме

Цель: Эхинококкоз — основной паразитический зооноз, представляющий проблему для системы здравоохранения в западной части Китая. По оценкам Министерства здравоохранения, в 2004 году данное заболевание было зафиксировано у 380 000 пациентов. На территории Цинхай-Тибетского нагорья успешно сосуществуют альвеолярный эхинококкоз (АЭ) и эхинококкоз мочевого пузыря (ЭМП). За последние годы китайское правительство увеличило объем финансирования мероприятий по контролю распространения данного заболевания в регионе. Таким образом, очень важно выявить значимые факторы риска заражения данной инфекцией путем анализа исследований, которые были проведены в регионе в течение последних десяти лет, с целью разработки соответствующих стратегий контроля сложившейся ситуации политиками.

Методология: Прежде всего, были определены критерии отбора анализируемой литературы. Был проведен систематический поиск публикаций, выпущенных за период с января 2000 года по июль 2011 года, в Medline, CNKI (Китайской национальной базе знаний) и Google Scholar. Затем были перечислены, посчитаны и обобщены значимые факторы риска, выявленные за счет одно- и/или многофакторного анализа. Сопоставимость данных была проверена путем изучения определенной литературы; возрастные и гендерные приоритеты в отношении аналогичных информационных структур объединили и использовали для дальнейшего анализа.

Результаты: На территории нагорья было проведено исследование огромного числа предполагаемых социальных, экономических, поведенческих и экологических факторов. В группе риска оказались тибетские пастухи, в особенности пожилые люди и женщины. Анализ объединенных сопоставимых данных показал, что число случаев заболевания среди женщин значительно выше, чем у других групп населения; кроме того, количество заболевших

эхинококкозом возрастает пропорционально возрасту пациента. Что касается поведенческих факторов риска, случаи заражения АЭ и ЭМП были, прежде всего, связаны с игрой с собаками, в то время как, с точки зрения гигиены, значимым фактором риска стало использование грунтовой воды в качестве питьевой. В качестве дефинитивных хозяев были определены, главным образом, собаки, выступающие носителями АЭ и/или ЭМП; лисы выступили как потенциальный фактор риска лишь в отношении АЭ. Выбивание пастбищ и уничтожение лесов сыграли свою роль только в случае заражения АЭ.

Заключение: Общины тибетских пастухов более других подвержены риску заражения эхинококкозом и должны контролироваться особым образом. Дегельминтизация как домашних, так и бродячих собак должна стать основным контролирующим мероприятием в отношении эхинококкоза; кроме того, необходимо рассмотреть лечение диких дефинитивных хозяев в зонах распространениях АЭ. Санитарное просвещение должно стать неотъемлемой частью системы обучения местного населения, ведь это единственный способ усовершенствовать существующие модели поведения. Требуется проведение дальнейших исследований с целью уточнения роли диких хозяев в процессе распространения АЭ/ЭМП, силы и объема воздействия изменений окружающей среды (выбивания пастбищ и уничтожения лесов) на распространение АЭ и факторов риска в Тибете.

Translated from English version into Russian by Irina Zayonchkovskaya, through

TRANSLATORS
WITHOUT BORDERS

Análisis de los factores de riesgo de prevalencia de la equinococosis humana en la meseta tibetana china: una perspectiva sobre las opciones de control

WANG Qian, HUANG Yan, HUANG Liang, YU Wenjie, HE Wei, L ZHONG Bo, LI Wei, ZENG Xiangman, Dominique A. VUITTON, Patrick GIRAUDOUX, Philip S. CRAIG, WU Weiping

Resumen

Objetivos: La equinococosis es una zoonosis parasitaria grave de relevancia para la salud pública en China occidental. El Ministerio de la Salud chino estimó en 2004 que 380.000 personas padecían la enfermedad en la zona. La meseta tibetana es una zona altamente endémica tanto de la equinococosis alveolar (AE) como de la equinococosis cística (CE). Durante los últimos años, el Gobierno chino ha estado aumentando la ayuda financiera para el control de las enfermedades en la zona. Por tanto, resulta de gran importancia identificar los factores de riesgo significativos de las enfermedades mediante el análisis de los estudios realizados en la zona durante la última década, con el fin de ayudar al los responsables de la toma de decisiones a diseñar las estrategias de control adecuadas.

Métodos: Se definieron en primer lugar los criterios de selección de la literatura a analizar. Medline, CNKI (China National Knowledge Infrastructure), y Google Académico se consultaron sistemáticamente para buscar literatura publicada entre enero de 2000 y julio de 2011. Se enumeraron, contaron y resumieron los factores de riesgo de riesgo significativos encontrados por análisis de factor único y/o múltiples factores. La literatura se estudió para comprobar la comparabilidad de los datos: las prevalencias específicas por edad y sexo con las mismas estructuras de datos se combinaron y utilizaron en análisis posteriores.

Resultados: Se estudió una variedad de supuestos factores de riesgo sociales, económicos, de conducta, y ecológicos en la meseta. Los pastores tibetanos, especialmente ancianos y mujeres, eran los que presentaban más riesgo. Mediante el análisis de datos comparables combinados, se averiguó que la prevalencia era sensiblemente superior en las mujeres, y que existía una linealidad positiva entre la prevalencia de la equinococosis y el envejecimiento. En cuanto a los factores de riesgo de conducta, en la mayoría de los casos existe una relación entre el juego con perros y la prevalencia de la CE y/o AE. En lo que respecta a la higiene, usar agua subterránea como fuente de agua potable estaba relacionado de forma significativa

con la prevalencia de la CE y la AE. Para el huésped definitivo, los factores asociados al perro se identificaron con más frecuencia con la prevalencia de la CE y de la AE. El zorro era un factor de riesgo potencial únicamente para la prevalencia de la AE. El sobrepastoreo y la deforestación resultaron significativos únicamente para la prevalencia de la AE.

Conclusión: Las comunidades de pastores tibetanos eran los que corrían un riesgo más elevado de prevalencia de equinococosis y deberían ser el objetivo del control de la enfermedad. La desparasitación, tanto de los perros domésticos como de los callejeros, debería ser una medida principal de control de la equinococosis. También debería tomarse en consideración el tratamiento de los huéspedes definitivos salvajes en zonas endémicas de AE. Las actividades de educación sanitaria deberían coincidir con el idioma y la base educativa de la población local, con el fin de poder mejorar las conductas. Se necesitan investigaciones posteriores para esclarecer la importancia de los huéspedes salvajes en la prevalencia de la AE/CE, el alcance y la amplitud de los impactos de los cambios ecológicos (sobrepastoreo y deforestación) en la prevalencia de la AE y los factores de riesgo en el Tíbet.

Translated from English version into Spanish by Raquel Bentué, through

TRANSLATORS WITHOUT BORDERS
